# Enhanced regional connectivity between western North American national parks will increase persistence of mammal species diversity

**DOI:** 10.1038/s41598-022-26428-z

**Published:** 2023-01-11

**Authors:** William D. Newmark, John M. Halley, Paul Beier, Samuel A. Cushman, Phoebe B. McNeally, Michael E. Soulé

**Affiliations:** 1 Natural History Museum of Utah, University of Utah, 301 Wakara Way, Salt Lake City, UT 84108 USA; 2 Department of Biological Applications and Technology, University of Ioannina, 45110 Ioannina, Greece; 3 School of Forestry and Merriam-Powell for Environmental Research, Northern Arizona University, Flagstaff, AZ 86011-5018 USA; 4 US Forest Service, Rocky Mountain Research Station, 2500 S Pine Knoll Dr, Flagstaff, AZ 86001 USA; 5 Department of Geography, University of Utah, Salt Lake City, UT 84112 USA; 6 P.O. Box 1801, Paonia, CO 81428 USA

**Keywords:** Ecological networks, Conservation biology

## Abstract

Many protected areas worldwide increasingly resemble habitat isolates embedded in human-modified landscapes. However, establishing linkages among protected areas could significantly reduce species-loss rates. Here we present a novel method having broad applicability for assessing enhanced regional connectivity on persistence of mammal diversity. We combine theoretically-derived species relaxation rates for mammal communities with empirically-derived pathways. We assess the value of enhanced regional connectivity for two hypothetical networks of national parks in western North America: the Yellowstone-Glacier network and the Mount Rainier-North Cascades network. Linking the Yellowstone and Glacier park assemblages by eliminating barriers to movement in identified mammal dispersal pathways and by incorporating adjacent wilderness areas and known ungulate migratory routes into a protected area network would greatly enlarge available habitat. This would enhance medium to large mammal species persistence time by factor of 4.3, on average, or ~ 682 generations relative to individual parks. Similarly, linking Mount Rainier and North Cascades park assemblages would enhance mammal species persistence time by a factor of 4.3, on average, or ~305 generations relative to individual parks. Enhancing regional connectivity among western North America parks could serve as an important template for landscape-scale conservation in the 21st century.

## Introduction

Protected areas are the cornerstone of biodiversity conservation worldwide. Yet the capacity of most protected areas to conserve biodiversity over the long-term is under threat from many factors including habitat loss and fragmentation, climate change, and over-exploitation of wildlife populations^1–6^. Of these threats, habitat loss and fragmentation on lands adjacent to protected areas are the most immediate and overarching threats facing most national parks and related reserves (IUCN protected area categories I & II) in western North America. As a result, most parks and related reserves in western North America are becoming increasingly spatially and functionally isolated in a matrix of human-altered habitats^1,3,7^. This is particularly problematic because few parks and related reserves worldwide are large enough to conserve intact plant and animal communities^8–11^ and many large-scale ecological processes, such as mammal migrations and disturbance regimes^12–16^. Consequently, there is an increasing effort worldwide to promote and establish protected area networks − networks of reserves interconnected by protected linkages^17,18^.

While the effectiveness of linkages or corridors in enhancing population persistence of species in habitat remnants is well-supported empirically, most studies that have demonstrated positive effects have been micro- or small-scale experiments or observational studies of single species^19^. There have been few assessments of the value of ecological linkages in enhancing species persistence at mid- to large spatial scales and at a community level^20,21^.

Most species extinctions in habitat remnants, including protected areas, following habitat loss are not immediate, but occur after a time lag^22,23^. The lag in species loss over time is because many species that occur in habitat remnants do not have viable populations. The delayed loss of species over time in habitat remnants is referred to as relaxation or faunal collapse^22^. The number or proportion of species that will eventually become extinct as a community reaches a new equilibrium is termed extinction debt^24,25^; with the time until one-half of all species that eventually will become extinct defined as the relaxation half-life^22,25^.

The delayed loss of species from habitat remnants following habitat loss offers an important, although largely unappreciated opportunity, to conserve species through targeted habitat protection and restoration^25,26^. Restoration of habitat, including the elimination of barriers to animal movement, can result in an increase in population size due to an expansion of habitat, and allows species to recolonize formerly occupied habitat remnants, and individuals to genetically and demographically rescue declining populations. The increase in relaxation time as a result of habitat restoration is referred to as species credit^27^.

Here we combine empirical relaxation half-life versus population capacity relationships for mammal communities in habitat remnants with multi-species empirically-derived pathways in two hypothetical protected area networks in western North America to assess the value of regional connectivity on the enhancement of species persistence – the tendency of species richness to persist. Specifically, we assess the increase in relaxation time and change in species number over time in protected area networks versus individual park/park assemblages. The hypothetical Yellowstone-Glacier and Mount Rainier-North Cascades protected area networks are presented as examples. This represents, as far as we are aware, the first application of relaxation theory to assess the value of enhanced regional connectivity in any proposed protected area network and provides a novel technique for assessing the value of enhanced regional connectivity among habitat remnants on persistence of mammal species diversity. We believe this analytical approach can assist conservation planners and decision makers in designing and evaluating regional-wide connectivity proposals and strategies.

We restrict the analysis to mammal species > 0.5 kg within the orders Artiodactyla, Carnivora, and Lagomorpha because of their lower densities and hence large area requirements^28^, and longer generation times^25^, and because sighting records at time of park establishment (*S*_*0*_) for these species are more complete due their larger body size and nonfossorial nature^1^. Additionally, these species have been the predominant focus of connectivity modeling and telemetric studies in these two networks.

An assumption of this analysis is that speciation and colonization are negligible within parks, which we believe is tenable. All of the parks/park assemblages included in this analysis have become over the last century increasingly spatially and functionally isolated for many medium to large mammal species as a result of anthropogenic activities and disturbance adjacent to the parks. These activities include highway, road and exurban development, predator control, hunting, trapping, logging, mining, grazing, and mechanized and non-mechanized recreation^8^, and vary by land ownership (public versus non-public) and management mandate (e.g., USFS national forests, wilderness areas). Indeed post-establishment patterns of extinctions and colonizations of medium to large mammals in 14 of the largest and oldest western North American parks, which also include the parks incorporated in this study, are consistent with the prediction that western North American parks are functionally analogous to land-bridge islands—islands that were formerly connected to a mainland and were created through a rise in sea level^1,8^.

## Results

### Relaxation half-life versus average population relation for mammals

**Figure 1:**
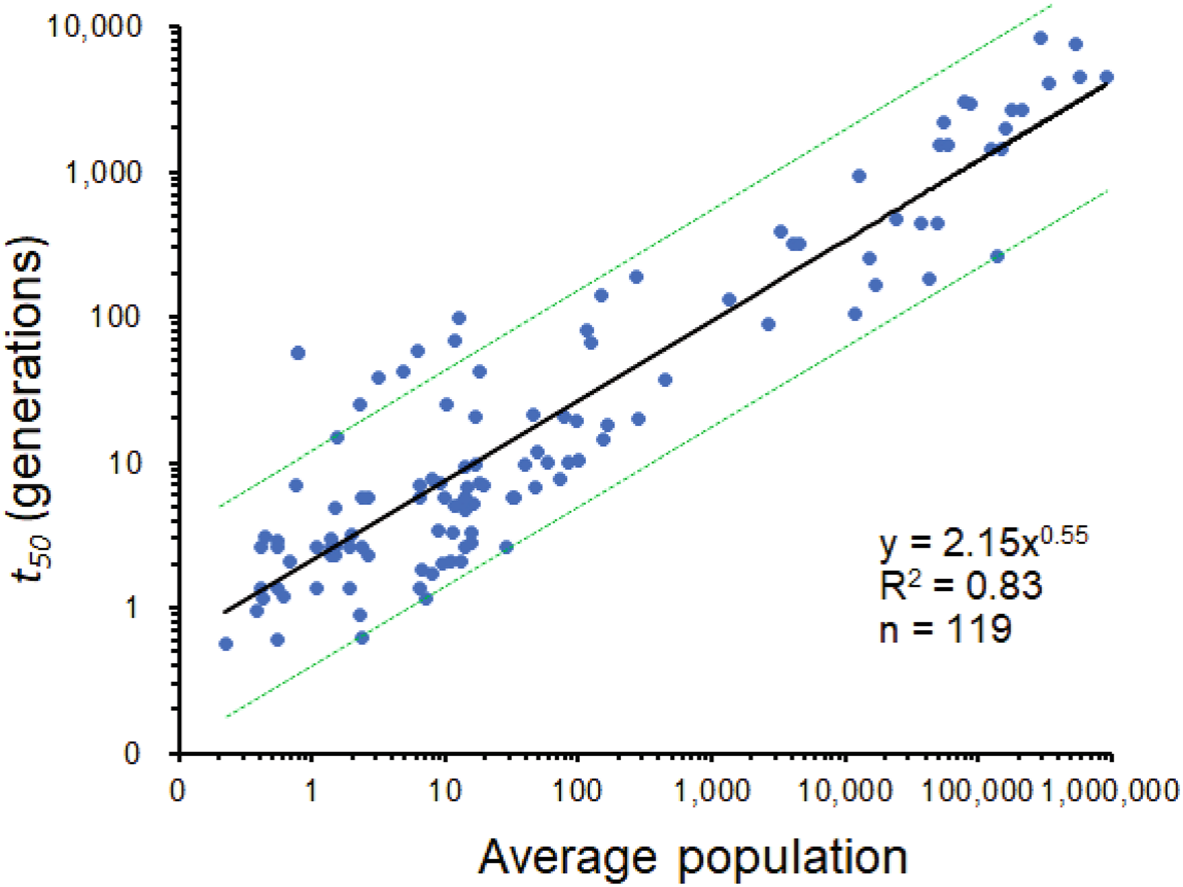
Relation between observed relaxation half-life (*t*_*5*0_) and average population per species for mammal communities in habitat remnants at the time of isolation. Data are from nine studies spanning four continents presented in a large meta-analysis by Halley et al. 2016^25^. The regression line is shown in black and the dashed green lines are the 90% prediction intervals. Prediction intervals were calculated in R^57^. The regression analysis estimates both the parameters of Eq. (5) (*c* = ln(2.15) and *α* = 0.55) and their standard errors (δ*c* = 0.134 and δ*α* = 0.023).

Assuming the decay of species number over time of the form Eq. (5) shown below, we estimate the observed relaxation half-life (*t*_50_) (in generations) for mammal communities (see Eq. (4)) to be:

1 $$t_{50} = 2.15n_{0}^{0.55}$$

where *n*_0,_ is average population per species at time of remnant isolation,

2 $$n_{0} = \frac{\rho A}{{S_{0} }}$$

and *ρ* is the total density of individuals in the community (Supplementary text), *A* is area (ha) and *S*_*0*_ is the initial number of species in the community. In a log-log regression, the log of average population explains 83% of the variation in log half-life for mammal communities in habitat remnants (Fig. 1). The relative uncertainty in Eq. (1) can be estimated, for these parameters, from Eq. (6) (see below) and is δ*t*_50_/*t*_50_ = 0.134 + 0.023ln(*n*_0_), using the results of the regression analysis depicted in Fig. 1 in which δ*c* = 0.134 and δ*α* = 0.023.

### Regional linkages

**Figure 2:**
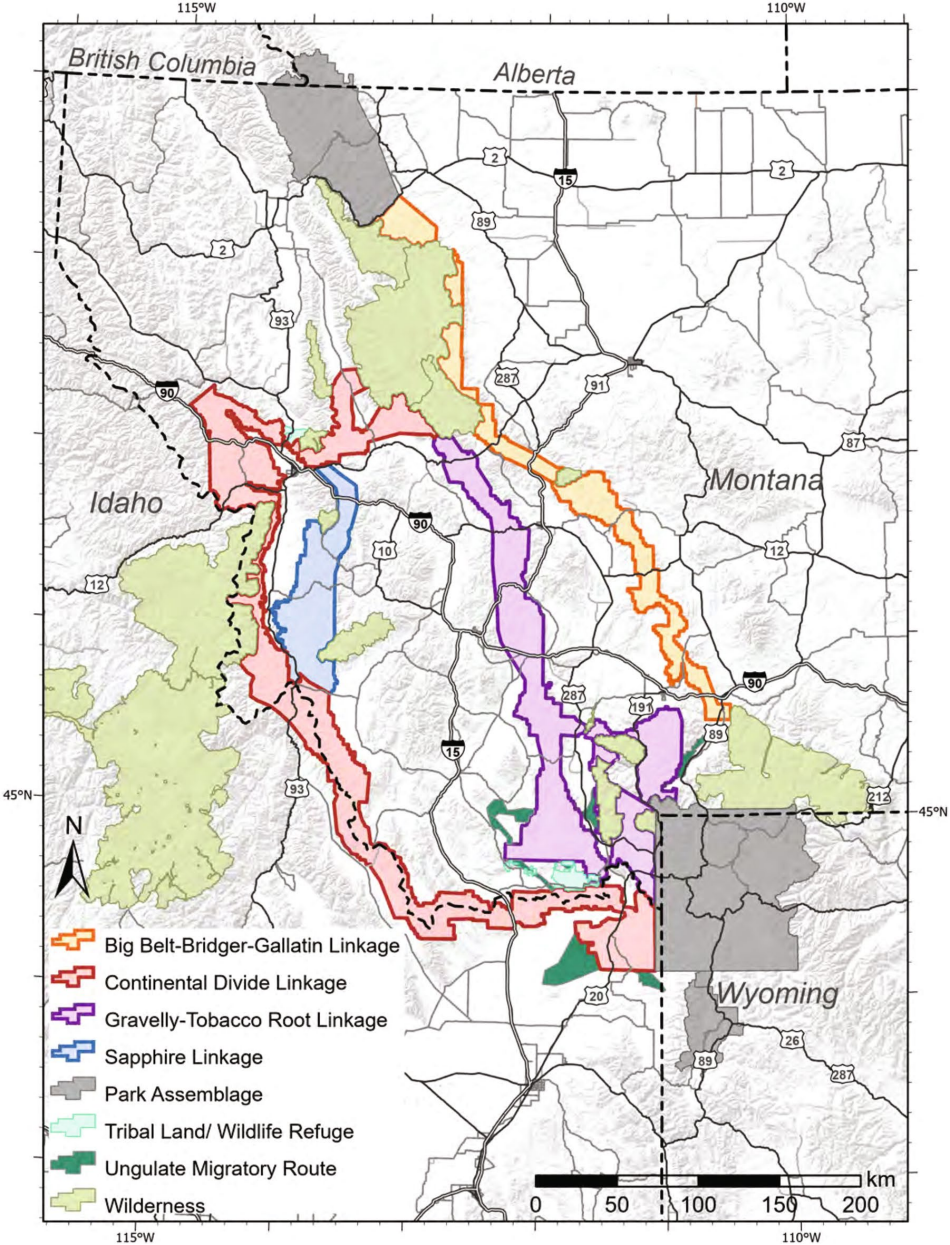
Location of identified multi-species linkages, and wilderness areas and ungulate migratory routes that intersect linkages between Yellowstone and Glacier park assemblages in the northern Rocky Mountains. The software used to create the maps in Figs. 2, 3 and 5 is ESRI ArcGis Pro V 2.7, https://www.esri.com/en-us/arcgis/products/arcgis-pro/overview.

Multi-species connectivity modeling for medium to large mammal species between Yellowstone and Glacier park assemblages (Supplementary Table S1)^29–35^ have identified four important linkages connecting these assemblages (Fig. 2). Telemetric studies of ungulate species have also identified seven multi- and single-species migratory routes within and adjacent to the Yellowstone park assemblage that intersect the four linkages^36^ (Fig. 2). An elimination of barriers to mammal movement within the four linkages in combination with protection of seasonal migratory routes intersecting the linkages as well as the incorporation of adjacent wilderness areas/protected areas also intersecting the linkages would create a functional protected area network of 7,428,131 ha (Table 1).

**Table 1:** Summary by area of park assemblages, linkages, and wilderness areas and seasonal migratory routes that intersect linkages in the Yellowstone-Glacier and Mount Rainier-North Cascades protected area networks.

Protected area network	Network element	Area (ha)
Yellowstone-Glacier	**Park assemblage**	
	Yellowstone-Grand Teton park assemblage	1,025,106
	Glacier-Waterton Lake park assemblage	457,883
	**Linkage**	
	Big Belt-Bridger-Gallatin mountain ranges	465,044
	Gravelly-Tobacco Root mountain ranges	885,535
	Continental Divide mountain range	1,246,362
	Sapphire mountain range	318,958
	**Wilderness areas adjacent to linkages**	
	Absorka-Beartooth, Anaconda-Pintler, Bob Marshall, Frank Church-River of No Return, Gates of the Mountain, Gospel-Hump, Great Bear, Lee Metcalf, Mission Mountain, Scapegoat, Rattlesnake, Selway- Bitterroot, Welcome Creek	2,848,431
	**Provincial parks, tribal lands, and national wildlife refuges adjacent to linkages**	
	Akaimina-Kishinena provincial park*,* Flathead reservation, Red Rocks national wildlife refuge	51,670
	Ungulate migratory routes that intersect linkages	129,142
	**Total**	7,428,131
Mount Rainier-North Cascades	**Park assemblage**	
	Mount Rainier national park	95,629
	North Cascades-Manning-Skagit park assemblage	314,565
	**Linkage**	
	north Cascades mountain range	1,064,495
	**Wilderness areas adjacent to linkage**	
	Alpine Lakes, Boulder River, Clearwater, Glacier Peak, Goat Rocks, Henry M Jackson, Lake Chelan- Sawtooth, Mount Baker, Noisy-Diobsud, Norse Peak, Pasayten, Tatoosh, Wild Sky, William O Douglas	979,124
	**Provincial parks and national recreation areas adjacent to linkage**	
	Cathedral provincial park/protected area, Chilliwack Lake provincial park, Snowy protected area, Lake Chelan national recreation area, Ross Lake national recreation area	141,004
	**Total**	2,594,819

**Figure 3:**
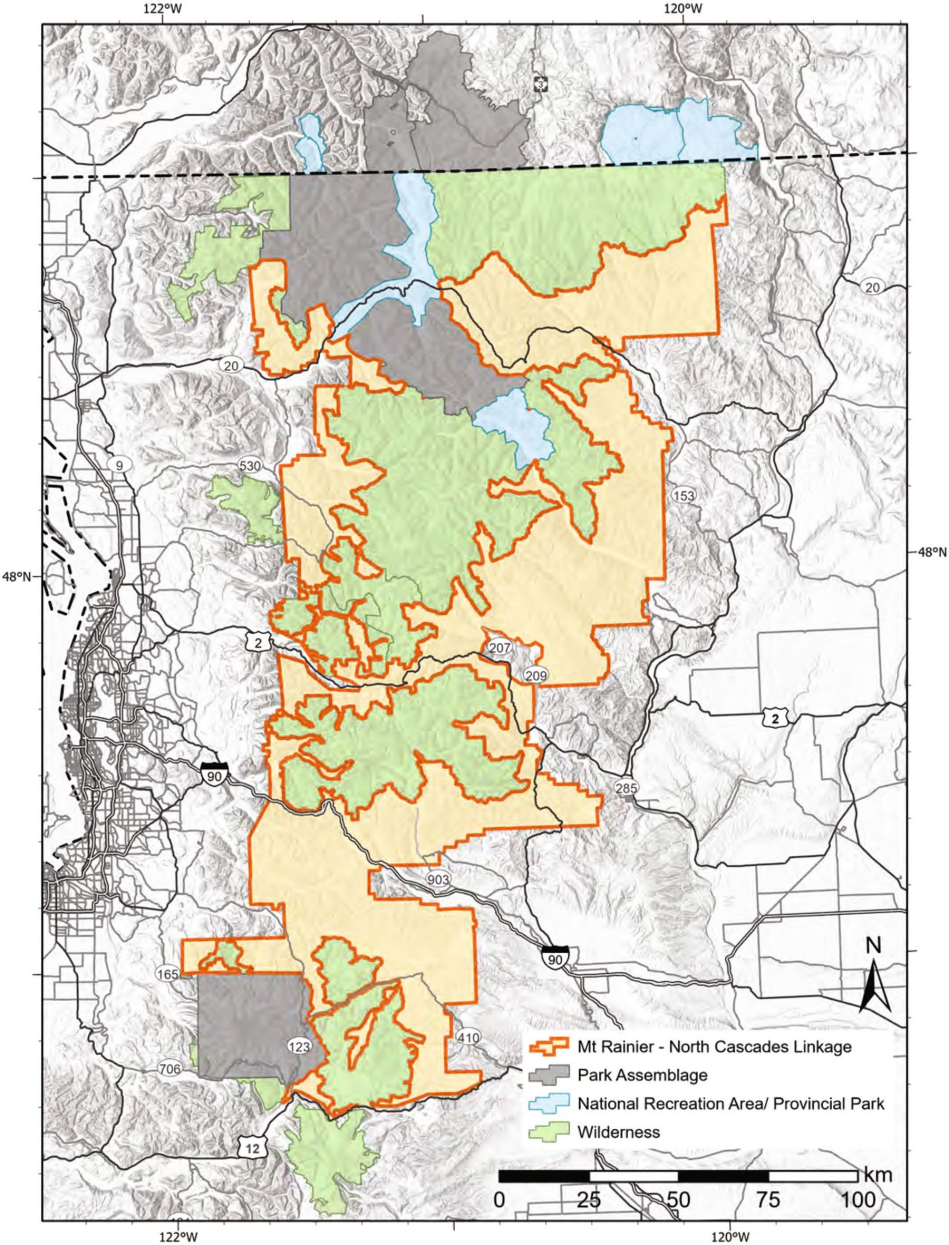
Location of identified multi-species linkage and wilderness areas that intersect the linkages between Mount Rainier National Park and the North Cascades park assemblage in the north Cascades mountain range.

Similarly, multi-species connectivity modeling of medium to large mammal movement between Mount Rainier and North Cascades park assemblages (Supplementary Table S1) has identified a single important linkage between park assemblages^37,38^ (Fig. 3). An elimination of barriers to mammal movement within this linkage and incorporating adjacent wilderness areas/protected areas would create a protected area network of 2,459,960 ha (Table 1).

### Impact of a protected area network on species credit

The relaxation half-life of medium to large mammal communities in the Yellowstone-Glacier protected area network is 905 generations in comparison to 280 generations in the Yellowstone park assemblage and 167 generations in the Glacier park assemblage. Elimination of impediments to mammal movement in the Yellowstone-Glacier protected area network would enhance mean (± SE) medium to large mammal species persistence (*t*_50_) by a factor of 4.3 (± 1.1) or ~ 682 generations in comparison to the individual park assemblages.

The relaxation half-life of medium to large mammal communities in the Mount Rainier-North Cascades protected area network is 411 generations in comparison to 141 generations in the North Cascades park assemblage and 71 generations in Mount Rainier national park. An elimination of barriers in the Mount Rainier-Glacier protected area network would enhance mean (± SE) medium to large mammal species persistence (*t*_50_) by a factor of 4.3 (± 1.4) or ~ 305 generations relative to the individual parks.

### Shorter-term extinction forecasts

**Figure 4:**
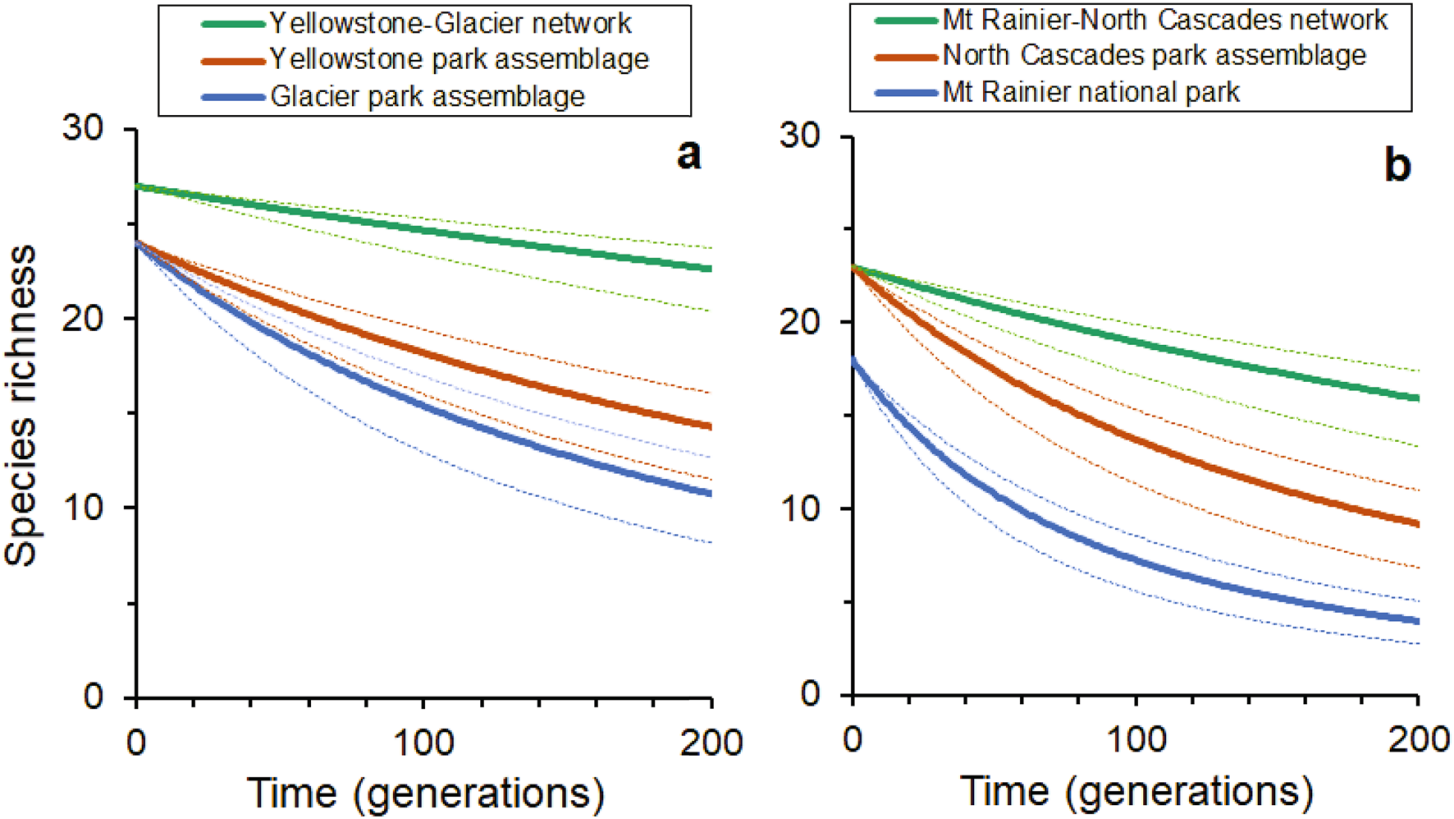
Comparison of projected change in medium to large mammal species number over 200 generations in (**a**) the Yellowstone-Glacier and (**b**) the Mount Rainier-North Cascades protected area networks with individual park/park assemblages. The upper and lower dashed lines are the loci of Eq. (6) for which *t*_50_ is replaced by *t*_50_ + δ*t*_50_ and *t*_50_− δ*t*_50_ respectively. These lines represent the uncertainty in predictions which reflect the fact that the model parameters are estimated from a large number of studies of mammals in different places at different times. Mean generation time (*τ*) in years for medium to large mammal species is presented by park assemblage and network in Supplementary Table 2.

Using the fitted parameters of Eq. (1) in Eq. (4) we find the observed relaxation half-life (*t*_50_) and hence parameterize Eq. (5) for individual parks/assemblages and park networks (Supplementary Tables S2 and S3) and use it to forecast the shorter-term relative loss of species richness over time. Projected change in species number over 200 generations are displayed in Fig. 4.

## Discussion

Ecologists have long promoted the enhancement of connectivity among habitat isolates to conserve species diversity in fragmented landscapes^39,40^. However, there have been few assessments of the value of enhanced ecological connectivity among habitat remnants on persistence of species diversity particularly at mid- to large spatial scales and at a community level^20,21^. Here we present a novel method for assessing the value of enhanced regional connectivity on persistence of species diversity by comparing persistence of mammal species diversity in two hypothetical protected area networks in western North America with that observed in individual park assemblages.

We believe this analytical approach is readily transferrable to other regions because relaxation theory is very general. This is illustrated in a large meta-analysis conducted by Halley et al.^25^ of the extinction dynamics of five major taxa (mammals, birds, reptiles, insects, plants) across multiple regions in the world. In this meta-analysis the relaxation parameters were what changed among taxa. Analyses of the extinction dynamics of birds^41^as well as an assessment of the value of targeted habitat restoration among forest fragments in reducing tropical bird species extinctions^21^ illustrate the transferability of this analytical approach to other taxa. Relaxation theory also incorporates dynamic equations which can be used to validate predicted change over time in species diversity in habitat remnants/protected areas. On the other hand, the generality of relaxation theory comes with a tradeoff in terms of reduced precision.

Practically, ecological benefits that accrue from enhanced connectivity between protected areas must inevitably be weighed against the social and economic costs associated with establishing such networks. In regions with high human population density enhancing connectivity among protected networks for taxa with large area requirements such as medium to large mammals can be challenging. Nonetheless, even in regions with high human population density we believe this new method of assessing the value of enhanced connectivity on persistence of mammal species diversity can be of considerable assistance to protected area planners and decision-makers in selecting and prioritizing the establishment of linkages between protected areas.

The analysis presented here reveals that enhancing regional connectivity between Yellowstone and Glacier, and Mount Rainier and North Cascades national park assemblages would greatly increase persistence or species credit for mammal species. The establishment of regional linkages would not only enhance movement and dispersal of species between park assemblages but combined with adjacent wilderness areas and ungulate migratory routes that intersect identified linkages would greatly enlarge existing protected areas and thus average population size of species. Enhancing regional connectivity between protected areas would increase medium to large mammal species persistence time by an average factor of 4.3 or ~ 682 generations within the Yellowstone-Glacier protected area network and by an average factor of 4.3 or ~ 305 generations years in the Mount Rainier-North Cascades protected area network.

Establishing regional connectivity among western North American parks would have a particularly pronounced and positive effect over the shorter-term in enhancing medium to large mammal species persistence in the smaller western North American parks (Fig. 4). For example, the enhancement in species number over 50 generations in a hypothetical Mount Rainier-North Cascades protected area network is relative to individual parks/park assemblages 3.0 times larger in Mount Rainier National Park – the smaller of the two park/park assemblages in this network – than in the larger North Cascades-Manning-Skagit park assemblage (Fig. 4).

Within the Yellowstone-Glacier and Mount Rainier-North Cascades protected area networks paved highways are an important barrier to mammal movement and dispersal^42^. Individual linkages within the two networks are bisected by 4–12, two- to four-lane highways (Supplementary Table S4). However, in recognition of the adverse impact of highways on mammal movement and dispersal, highway authorities are beginning to construct wildlife crossings − over- and under-passes combined with continuous fencing along highway edges − along sections of highways within the two networks^38,43–45^. Over the last two decades, 39 under- and over-passes have been constructed along a 90 km stretch of Highway 93 on the Flathead Indian Reservation west of Glacier National Park^45^. Cushman et al.^42^ have also identified and ranked more than 190 additional highway crossing locations for black bear in proposed linkages between Yellowstone and Glacier national park assemblages. Similarly more than 30 under- and over-passes have been constructed along a 24 km stretch of Interstate 90 near Snoqualmie Pass north of Mt Rainier National Park^46^.

Post-construction monitoring of wildlife crossings in the Yellowstone-Glacier and Mount Rainier-North Cascades protected area networks have documented multiple mammal species using the crossings^44,45^. Yet an expanded number of wildlife crossings will certainly be required, particularly given the pernicious impacts of road networks on mammal populations^47^.

Population-level monitoring of wildlife crossings is essential to assess their effectiveness^48,49^. Such a program is being implemented within and adjacent to a number of protected areas in western North America. Studies in Banff National Park have found that wildlife crossings over the Trans Canadian Highway do provide genetic connectivity for black and grizzly bears^50^_,_ and wolverine, although in the case of the latter species for only males^51^_._ The apparent reluctance of female wolverine to use wildlife crossings would appear to be related more to the location of the crossing or possibly a longer learning time rather than an inherent inability to use wildlife crossings^51^.

In the northern Rockies and north Cascades, housing development is an additional impediment to mammal movement. Between 1940 and 2000, 28 million housing units were built in the United States within 50 km of protected areas^3^. Seventeen million housing units are predicted to be built within 50 km of protected areas by 2030^3^. In the vicinity of Yellowstone, Glacier, Mount Rainier, and North Cascades national parks, housing growth rates between 1940 and 2000 on non-publicly managed lands have been among the highest adjacent to protected areas nationwide, with rates > 300–400%^3^. Yet, fortunately, non-publicly managed/tribal lands comprise on average (mean ± SD) < 4% (3.9 ± 0.3 %) of the total area of the two hypothetical protected area networks. Establishment of conservation easements, land trusts, and public and private land swaps and purchases, in combination with land use zoning, are approaches that have been successfully used in western North America and elsewhere to conserve critical wildlife habitat^36,52^ and may be essential to preventing the blockage of critical “chokepoints” within linkages in the two protected area networks.

Additionally, non-physical anthropogenic barriers that alter or prevent mammal dispersal and movement through identified linkages on public lands would need to be carefully managed. Seasonal closures, user quotas and regulations, and restrictions on mechanized and non-mechanized recreation are approaches that public land-managing agencies are currently implementing to protect wildlife and critical habitat in portions of the identified linkages.

Enhancing regional connectivity among national parks in western North America would also permit plant and animal species to more readily shift their geographic ranges in response to climate change. Although there have been few studies that have examined the impact of climate change on dispersal and movement of mammals among protected areas in western North America^32,53,54^, an important example is work by McKelvey et al.^32^ who examined the impact of climate change in the central and northern Rockies and north Cascades on wolverine dispersal and distribution. Based on projected distribution of snow cover through May 15, which coincides with the end of the denning period, they found that least-cost pathways for wolverine in the Yellowstone-Glacier protected area network are predicted to shift eastward over this century from the current Continental Divide and Sapphire mountain ranges to a more north-south connection following the Gravelly-Tobacco Root mountains ranges (Fig. 2). This finding highlights the importance and value of protecting multiple linkages in a protected area network.

A concerted effort will be required to enhance the capacity of national parks and related reserves in western North America to conserve intact plant and animal communities over the coming century. Implementing a regional-wide program to establish linkages among national parks and related reserves in western North America, including Yellowstone National Park, North America’s first national park, would greatly enhance the persistence of plant and animal communities in the northern Rockies and north Cascades. Programs to enhance regional connectivity among protected areas are being initiated elsewhere in western North America (e.g., Yellowstone to Yukon Project) as well as on five other continents^18^. Thus, the enhancement of regional connectivity in the Yellowstone-Glacier and Mount Rainier-North Cascade networks could serve as an important template for not only the recent 30 × 30 initiative in the United States but landscape scale conservation in the 21st century in general.

## Methods

**Figure 5:**
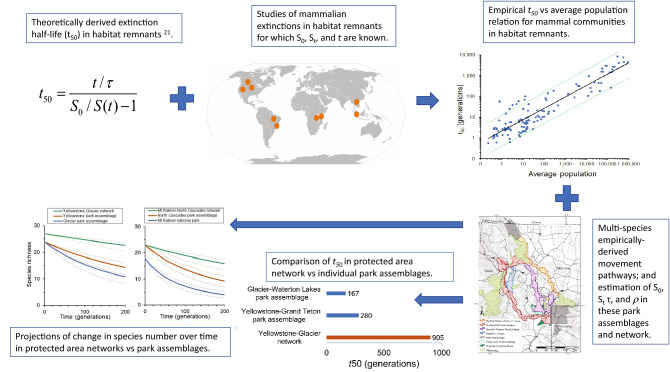
A methodological workflow.

A methodological workflow is presented in Fig. 5.

### Study sites

We selected two proposed protected area networks in western North America, the “Yellowstone-Glacier” and “Mount Rainier-North Cascades” networks, to assess the value of regional connectivity on enhancement of persistence of medium to large mammal species. We selected these two networks for several reasons. First, researchers have previously modeled empirically-derived movement pathways for multiple mammal species between individual parks or across regional landscapes in which the parks are embedded (Supplementary Table S1). Secondly, there is detailed knowledge about the location of seasonal migratory routes for multiple ungulate species within and adjacent to several of the parks (Yellowstone and Grand Teton national parks)^36^. Thirdly, the location of physical barriers to movement (roads, highways, exurban development), in relation to least-cost pathways and migratory routes for mammal species are well-defined^36,55^. Fourthly, there is increasing experience and knowledge in these two protected area networks about mitigating impacts of highways and roads on mammal movement through the construction of under- and over-passes^36,38,43–45^. Lastly, identified linkages within these two networks occur predominantly on publicly-managed lands, and thus enhancing regional connectivity in these two networks is considerably more feasible than in many other regions in western North America.

In this analysis, we defined two or more contiguous parks as a single park assemblage. Yellowstone and Grand Teton national parks, and Glacier and Waterton Lakes national parks are defined as single park assemblages in the Yellowstone-Glacier protected area network; and North Cascades national park and Manning and Skagit provincial parks are defined as a single park assemblage in the Mount Rainier-North Cascades protected area network.

### Estimation of half-life of a habitat remnant

In a large meta-analysis, Halley et al.^25^ documented the dynamics of extinction debt of species in habitat remnants across multiple taxa including mammals, birds, reptiles, insects, and plants, using a model of community dynamics. Here we utilize empirical estimates of extinction half-life for mammal communities that are derived from 9 studies spanning four continents to estimate an extinction half-life versus average population per species (namely *n*_0_, see Eq. (2)) relationship for mammals. In this compilation, we excluded the four national parks/park assemblages that are incorporated in this study^8^. In the relaxation process, the half-life is the time for species richness to fall to half of its initial value (S_0_). Typically, for each habitat remnant, *k*, we have an observation of species richness at time *t* after habitat loss *S*_*k*_(*t*) relative to its initial value *S*_*k*_(0). Each remnant has a half-life *t*_*50*_, which we can estimate using the neutral-theory formula^41^ and that assumes species are equivalent, and community dynamics are driven by demographic stochasticity, and negligible dispersal and speciation:

3 $$t_{50} = \frac{t/\tau }{{S_{0} /S(t) - 1}}$$

which is a good approximation for the number of generations to fall to half its initial value^25^ and where *τ* is mean generation time. It can be shown that the corresponding expected half-life (also in generations) from theoretical considerations using a model^25^ has the form:

4 $$t_{50} = Cn_{0}^{\alpha }$$

Parameters *α* and *C* represent the slope and intercept of the regression line in Fig. 1. The slope *α* may be interpreted as a measure of how sharply persistence time (*t*_*50*_) increases with the population per species (*n*_*0*_), while *C* marks the persistence time when *n*_0_ = 1. Thus, a set of observations $n_{0}^{(1)} ,n_{0}^{(2)} ,\;...,n_{0}^{(K)} \;$ associated with $t_{50}^{(1)} ,\;t_{50}^{(2)} ,\;...,t_{50}^{(K)} \;$ respectively can be used to fit a least-squares line on log-scale, and to find *α* and *C*. From *C* we can find *k* using Eq. (4). Although *C* is not independent of *α*, so it is best to use a nonlinear solution procedure to estimate the parameters *α* and *C*, the values found differ little from those of a simple regression fit in Fig. 1.

An estimate of the error associated with this formula is found from the error in the simple regression model. This model offers two error estimates for each of the two fitted parameters: *α* and ln*C*. Each of which has large errors due to the highly scattered nature of the data (Fig. 1). If we carry out regression in the log domain with *y* = ln(*t*_50_), *x* = ln(*n*_0_) and *c* = ln(*C*) we fit the equation

5 $$y = c + \alpha x$$

The regression analysis gives us uncertainties in the estimated parameters with their associated uncertainties: *c* + δ*c* and *α* + δ*α*. Exponentiating (5) gives Eq. (4), from which we can derive the equation for the uncertainty:

6 $$\delta t_{50} \quad = \quad \frac{{\partial t_{50} }}{\partial c} \cdot \delta c + \frac{{\partial t_{50} }}{\partial \alpha } \cdot \delta \alpha \quad = \quad \left[ {\delta c\quad + \quad \ln (n_{0} ) \cdot \delta \alpha } \right] \cdot t_{50}$$

This expected uncertainty in the half-life is typically large (see Fig. 4), since it reflects the fact that the basic parameterization of the model is based on a range of studies across a wide range of places at different times.

### Modeling least cost movement pathways within protected area networks

Workers have previously identified movement pathways for multiple mammal species between park/park assemblages in the Yellowstone-Glacier and Mount Rainier-North Cascades protected area networks using predominantly least-cost pathway analysis (Supplementary Table S1). A least-cost pathway or corridor is a swath of cells predicted to provide the lowest cumulative cost of movement between a pair of polygons, in this case national parks, which are referred to as termini so as to avoid arbitrarily labeling one as source and the other as target^30,56^.

The first step in defining a least-cost pathway or corridor is to define the analysis area which includes not only the land between the termini, but also a larger buffer, so that widely looping low cost corridors can be discerned. Next a map of resistance values for all cells is developed for the analysis area. The resistance value for each cell is species-specific; it reflects the energy cost, mortality risk, or movement difficulty for an individual of that species to cross the cell, estimated as a function of cell attributes such as land cover, land use, topography, and proximity to features such as highways or open water. The relationship between cell attributes and resistance can be derived in several ways; in order of increasing rigor and relevance, these include expert opinion, habitat use, animal movement in the home range, animal movement during migration or dispersal, and gene flow. Next a cost map is generated from the resistance map, where the cost value for each cell is the smallest possible sum of resistances along a path between the two termini, with the constraint that the path must go through the focal cell. Workers typically identify all cells with cost below several maximum values (e.g., 1%, 1.5%, 2% of the analysis area), and select the maximum value for which the resulting swath of low cost cells has an acceptable minimum and average width. In some cases this swath of cells consist of a single strand, but can also diverge into more than one strand for part or all of its length. The resulting map indicates routes or zones that would permit the most efficient movement for each focal species. The final step is to join the least cost corridors for the focal species into an overall connectivity zone. Here we present a union of single-species least cost pathways combined with observed migratory movement paths.

### Linkage boundaries

Identified least-cost pathways or linkages in the Yellowstone-Glacier and Mount Rainier-North Cascades protected area networks predominantly follow one or more mountain ranges. Linkage boundaries were defined by projecting over a least cost pathway a ~ 5 – 45 km buffer which largely followed existing boundaries of publicly-managed lands, because we believe these are the widest practical boundaries that can be easily established in the two hypothetical protected area networks. Urban and exurban development that intersected linkage boundaries were excluded. Additionally in defining the boundaries of a modeled pathway or linkage, we explicitly incorporated a broad elevational gradient of publicly-managed lands, predominantly US Forest Service national forests, along the identified pathway because modeling of regional connectivity across the Northern Rockies for a set of hypothetical species that vary in their resistance surfaces and dispersal abilities has previously highlighted the importance of particularly low-elevation habitat in maximizing community-wide connectivity^55^. We have also included in defining the boundaries of a linkage known migratory routes − pathways that link winter and summer range for ungulates and intersect a linkage.

### Species richness at time of park and network establishment (***S***_***0***_)

Species richness at time of park establishment (*S*_*0*_) was calculated from historical surveys and sighting records for individual parks (Supplementary Table S2)^1,8^. In this analysis, we assumed initial species richness (*S*_*0*_) in a protected area network was total species richness across all individual parks/park assemblages at time of establishment within a network (*S*_*0*_). We also assumed in calculating the extinction half-life (*t*_*50*_) and relaxation rates of mammal communities in protected area networks an absence of barriers to mammal movement.

### Change in species number over time

If we assume that speciation and immigration are negligible, following the methods of Halley et al.^25^ the number of species in an isolated fragment, as a function of time^21^, is as follows:

7 $$S(t) = \frac{{S_{0} }}{{\left[ {1 + (2^{\alpha } - 1)\frac{t}{{t_{50} }}} \right]^{1/\alpha } }}$$

Here, *S*_*0*_ is initial species richness, and *t* is length of time in years between *S*_*0*_ and *S*(*t*). This decay is also a function of two parameters: the half-life *t*_50_ for each habitat remnant and the parameter *α* that is the same for all habitat remnants.

## Supplementary Information

Supplementary Information.

## Data Availability

All data are available in the main text and supplementary information.
