# Supplementary material for: Enhanced regional connectivity between western North American national parks will increase persistence of mammal species diversity
Source: Sci Rep. 2023 Jan 11;13:474. doi: 10.1038/s41598-022-26428-z (PMC9834291; doi:10.1038/s41598-022-26428-z)
Supplement: Supplementary file 1 — Supplementary Information. [file 41598_2022_26428_MOESM1_ESM.docx]

Supplementary Information

**Enhanced regional connectivity between western North American national parks will increase persistence of mammal species diversity**

William D. Newmark, John M. Halley, Paul Beier, Samuel A. Cushman, Phoebe B. McNeally, and Michael E. Soulé

Supplementary Text

**Estimation of species-specific population density and generation time**

Population density of medium to large mammal species in northwestern North America was taken from the literature ^1–41^ and corrected for available habitat within a park/park assemblage or network. Available habitat was defined in terms of a species’ predominant habitat associations, which was taken from the literature ^42^, and then classified in terms of 15 major North American land cover types mapped at a spatial resolution of 30 m ^43^. Species-specific available habitat within a park/park assemblage or network was estimated by projecting the land cover types over individual park/park assemblages and networks and calculating the percent cover in ArcGIS. Species-specific generation time for large mammals was based on allometric relations between body mass ^42^ and generation time ^44^. Total density of individuals per hectare and mean generation time of medium to large mammals by protected area network and park/park assemblage are presented in Supplementary Table S2; and medium to large species occurring at time of protected area establishment, and species-specific density and generation time by park/park assemblage and protected area network are presented in Supplementary Table S3.

**Supplementary Table S1**. Focal species, location of modeled pathway, and methodological approach adopted in the pathway analysis within the proposed Yellowstone-Glacier and Mount Rainier-North Cascades protected area networks.

| **Protected area network** | **Focal Species** | **Location of modeled pathway** | **Methodological Approach** | **Source** |
| --- | --- | --- | --- | --- |
| Yellowstone-Glacier | Gray Wolf | Big Belt-Bridger-Gallatin; Continental Divide; Gravelly-Tobacco Root mountain ranges | Logistic regression habitat selection models | Oakleaf et al. 2006 ^45^ |
|  | Black Bear | Big Belt-Bridger-Gallatin; Gravelly-Tobacco Root mountain ranges | Least-cost pathway analysis | Cushman et al. 2009 ^46^ |
|  | Wolverine | Continental Divide mountain range | Least-cost pathway analysis and electrical circuit theory | Schwartz et al. 2014 ^47^ |
|  | Wolverine | Continental Divide-Sapphire mountain ranges* | Least-cost pathway analysis | McKelvey et al. 2011 ^48^ |
|  | Gray Wolf | Continental Divide mountain range† | Least-cost pathway analysis and electrical circuit theory | Carroll et al. 2012 ^49^ |
|  | Wolverine | Continental Divide-Sapphire mountain ranges‡ | Least-cost pathway analysis under different weighting schemes | Parks et al. 2013 ^50^ |
|  | Grizzly Bear | Big Belt-Bridger-Gallatin mountain ranges§ | Randomized shortest path | Peck et al. 2017 ^51^ |
| Mount Rainier-North Cascades | Wolverine | north Cascades mountain range | Least-cost pathway analysis | Singleton et al. 2002 ^52^ |
|  | Gray Wolf | north Cascades mountain range | Least-cost pathway analysis | Singleton et al. 2002 ^52^ |
|  | Grizzly Bear | north Cascades mountain range | Least-cost pathway analysis | Singleton et al. 2002 ^52^ |
|  | Lynx | north Cascades mountain range | Least-cost pathway analysis | Singleton et al. 2002 ^52^ |
|  | Marten | north Cascades mountain range | Landscape genetic methods | Long et al. 2013 ^53^ |
|  | Black Bear | north Cascades mountain range | Landscape genetic methods | Long et al. 2013 ^53^ |

* McKelvey et al. (2011) modeled current and future least-cost pathways between Yellowstone and Glacier national parks under various climate change scenarios. The Continental Divide-Sapphire mountain ranges were identified as the least-cost pathway under current climatic conditions.

† Carroll et al. (2012) modeled least-cost and electrical resistance pathways between Yellowstone and the central Idaho wilderness complex.

‡ Parks et al. (2013) modeled least-cost pathways under different weighting schemes. Results presented here are for least-cost pathways modeled under an assumption of exponential decay weighting for purposes of connecting populations demographically.

§ Peck et al. (2017) modeled pathways using randomized shortest path analysis. Although multiple pathways between the Yellowstone Ecosystem and the northern Continental Divide ecosystem were identified, the most predominant pathway between Yellowstone and Glacier national parks followed the Big Belt-Bridger-Gallatin mountain ranges.

**Supplementary Table S2**. Number of species >0.5 kg of lagomorph, carnivore, and artiodactyl at time of establishment (*S_0_*), total density of individuals across all species per hectare (*ρ)*, and mean generation time (*τ*) of medium to large mammal species. Species richness in protected area networks at time of establishment (*S_0_*) is assumed to be total species richness across all individual parks/park assemblage within a network.

| **Protected area network**  **park assemblage** | **Species richness at time of establishment (*S_0_*)** | ***S_0_* source** | **Total density of individuals per ha** **(*ρ)*** | **Mean generation time (*τ*) in years** |
| --- | --- | --- | --- | --- |
| Yellowstone-Glacier network | 27 | ^54–61^ | 0.215 | 4.6 |
| Yellowstone-Grand Teton park assemblage | 24 | ^55,58,59^ | 0.164 | 4.3 |
| Glacier-Waterton Lake park assemblage | 24 | ^54,56,57,60,61^ | 0.143 | 4.4 |
|  |  |  |  |  |
| Mount Rainier-North Cascades network | 23 | ^62–69^ | 0.125 | 4.2 |
| Mount Rainier national park | 18 | ^62,65–67,69^ | 0.109 | 3.4 |
| North Cascades-Manning-Skagit park assemblage | 23 | ^63,64,68,69^ | 0.147 | 4.2 |
|  |  |  |  |  |

**Supplementary Table S3**. Medium to large mammal species > 0.5 kg of lagomorph, carnivore, and artiodactyl occurring at time of park/park assemblage and protected area network establishment; and species-specific density (per ha) and generation time (in years) within a park/park assemblage and protected area network.

| **Species** | **Mt Rainier national park** | | **North Cascades park assemblage** | | **Mt Rainier – North Cascades network** | | **Yellowstone – Grand Teton park assemblage** | | **Glacier-Waterton Lakes park assemblage** | | **Yellowstone-Glacier network** | |
| --- | --- | --- | --- | --- | --- | --- | --- | --- | --- | --- | --- | --- |
|  | **density** | **Gen. time** | **density** | **Gen. time** | **density** | **Gen. time** | **density** | **Gen. time** | **density** | **Gen. time** | **density** | **Gen. time** |
| **Lagomorph** |  |  |  |  |  |  |  |  |  |  |  |  |
| Nuttall's cottontail *Syvilagus nuttallii* |  |  |  |  |  |  | 0.0007 | 1.6 |  |  | 0.0033 | 1.6 |
| Snowshoe hare *Lepus americanus* | 0.0586 | 1.9 | 0.0540 | 1.9 | 0.0586 | 1.9 | 0.0419 | 1.9 | 0.0592 | 1.9 | 0.0476 | 1.9 |
| White-tailed jackrabbit *Lepus townsendii* |  |  |  |  |  |  | 0.0057 | 2.3 | 0.0080 | 2.3 | 0.0096 | 2.3 |
| **Carnivore** |  |  |  |  |  |  |  |  |  |  |  |  |
| Coyote *Canis latrans* | 0.0027 | 3.5 | 0.0029 | 3.5 | 0.0025 | 3.5 | 0.0040 | 3.5 | 0.0033 | 3.5 | 0.0043 | 3.5 |
| Gray wolf *Canis lupus* | 0.0001 | 4.6 | 0.0001 | 4.6 | 0.0001 | 4.6 | 0.0001 | 4.6 | 0.0001 | 4.6 | 0.0002 | 4.6 |
| Red fox *Vulpes vulpes* | 0.0001 | 2.7 | 0.0001 | 2.7 | 0.0000 | 2.7 | 0.0001 | 2.7 | 0.0001 | 2.7 | 0.0001 | 2.7 |
| Black bear *Ursus americanus* | 0.0003 | 5.6 | 0.0003 | 5.6 | 0.0003 | 5.6 | 0.0004 | 5.6 | 0.0006 | 5.6 | 0.0006 | 5.6 |
| Grizzly bear *Ursus arctos* |  |  | 0.00001 | 7.9 | 0.00001 | 7.9 | 0.0001 | 7.9 | 0.0002 | 7.9 | 0.0001 | 7.9 |
| Raccoon *Procyon lotor* | 0.0058 | 2.9 | 0.0063 | 2.9 | 0.0054 | 2.9 |  |  |  |  |  |  |
| Wolverine *Gulo luscus* | 0.00003 | 3.9 | 0.00003 | 3.9 | 0.00003 | 3.9 | 0.00002 | 3.9 | 0.00003 | 3.9 | 0.00002 | 3.9 |
| River otter *Lutra canadensis* | 0.00003 | 3.2 | 0.00003 | 3.2 | 0.00003 | 3.2 | 0.0001 | 3.2 | 0.0001 | 3.2 | 0.00003 | 3.2 |
| Marten *Martes americana* | 0.0016 | 1.6 | 0.0016 | 1.6 | 0.0017 | 1.6 | 0.0027 | 1.6 | 0.0013 | 1.6 | 0.0037 | 1.6 |
| Fisher *Martes pennanti* | 0.0008 | 2.5 | 0.0008 | 2.5 | 0.0008 | 2.5 |  |  | 0.0007 | 2.5 | 0.0007 | 2.5 |
| Striped skunk *Mephitis mephitis* | 0.0194 | 2.4 | 0.0210 | 2.4 | 0.0181 | 2.4 | 0.0224 | 2.4 | 0.0209 | 2.4 | 0.0239 | 2.4 |
| Mink *Mustela vison* | 0.0004 | 1.8 | 0.0001 | 1.8 | 0.0004 | 1.8 | 0.0024 | 1.8 | 0.0018 | 1.8 | 0.0006 | 1.8 |
| Spotted skunk *Spilogale putorius* | 0.0170 | 2.3 | 0.0184 | 2.3 | 0.0159 | 2.3 |  |  |  |  |  |  |
| Badger *Taxidea taxus* |  |  | 0.0106 | 3.0 | 0.0121 | 3.0 | 0.0044 | 3.0 | 0.0156 | 3.0 | 0.0217 | 3.0 |
| Mountain lion *Felis concolor* | 0.0001 | 5.2 | 0.0001 | 5.2 | 0.0001 | 5.2 | 0.0002 | 5.2 | 0.0006 | 5.2 | 0.0002 | 5.2 |
| Lynx  *Lynx canadensis* | 0.0002 | 3.2 | 0.0002 | 3.2 | 0.0002 | 3.2 | 0.0001 | 3.2 | 0.0001 | 3.2 | 0.0001 | 3.2 |
| Bobcat *Lynx rufus* | 0.0014 | 3.5 | 0.0015 | 3.5 | 0.0013 | 3.5 | 0.0002 | 3.5 | 0.0002 | 3.5 | 0.0002 | 3.5 |
| **Artiodactyla** |  |  |  |  |  |  |  |  |  |  |  |  |
| Moose *Alces alces* |  |  | 0.0006 | 8.9 | 0.0007 | 8.9 | 0.0017 | 8.9 | 0.0007 | 8.9 | 0.0011 | 8.9 |
| Wapiti *Cervus elaphus* | 0.0001 | 5.2 | 0.0001 | 5.2 | 0.0001 | 5.2 | 0.0002 | 5.2 | 0.0006 | 5.2 | 0.0002 | 5.2 |
| Mule deer *Odocoileus hemionus* | 0.0002 | 5.4 | 0.0002 | 5.4 | 0.0002 | 5.4 | 0.0146 | 0.6 | 0.0005 | 5.4 | 0.0156 | 5.4 |
| White-tailed deer *Odocoileus virginianus* |  |  |  |  |  |  | 0.0001 | 6.1 | 0.0030 | 6.1 | 0.0042 | 6.1 |
| Caribou *Rangifer tarandus* |  |  |  |  |  |  |  |  | 0.0002 | 7.0 | 0.0002 | 7.0 |
| Bison *Bison bison* |  |  |  |  |  |  | 0.0042 | 10.4 |  |  | 0.0071 | 10.4 |
| Mountain goat *Oreamnos americanus* | 0.0008 | 5.5 | 0.0007 | 5.5 | 0.0003 | 5.5 |  |  | 0.0007 | 5.5 | 0.0001 | 5.5 |
| Mountain sheep *Ovis canadensis* |  |  | 0.0002 | 6.5 | 0.0001 | 6.5 | 0.0001 | 6.5 | 0.0003 | 6.5 | 0.0002 | 6.5 |
| Pronghorn *Antilocapra americana* |  |  |  |  |  |  | 0.0002 | 4.9 |  |  | 0.0012 | 4.9 |

**Supplementary Table S4**. Two and four-lane pave highways that bisect regional linkages in the Yellowstone-Glacier and Mount Rainier-North Cascades protected area networks.

| **Protected Area Network** | **Regional Linkage** | **Two and four-lane bisecting highways** |
| --- | --- | --- |
| Yellowstone-Glacier | Big Belt-Bridger-Gallatin mountain ranges | US89, I90, MT86, US12, I15, MT200,US 2 |
|  | Gravelly-Tobacco Root mountain ranges | US20, MT87, US287, US191, MT55, I90, MT2, MT69, US55, I15,US12, MT200 |
|  | Continental Divide mountain range | US20, I15, MT43,US93,US12, I90, MT200, MT83 |
|  | Sapphire mountain range | US93, MT38, I90, MT200 |
|  |  |  |
| Mount Rainier – North Cascades | north Cascades mountain range | I90, US2, US20, US97, WA410, WA530, WA903, WA123, WA706, WA165, WA131, US12 |

­

**Supplementary References**

1. Gilbert, B. A. & Raedeke, K. J. Recruitment dynamics of black-tailed deer in the Western Cascades. *J. Wildl. Manage.* **68**, 120–128 (2004).

2. Knick, S. T., Brittell, J. D. & Sweeney, S. J. Population characteristics of bobcats in Washington state. *J. Wildl. Manage.* **49**, 721–728 (1985).

3. Eisenberg, C., Hibbs, D. E., Ripple, W. J. & Salwasser, H. Context dependence of elk (Cervus elaphus) vigilance and wolf (Canis lupus) predation risk. *Can. J. Zool.* **92**, 727–736 (2014).

4. Kunkel, K. E., Pletscher, D. H., Boyd, D. K., Ream, R. R. & Fairchild, M. W. Factors correlated with foraging behavior of wolves in and near Glacier National Park, Montana. *J. Wildl. Manage.* **68**, 167–178 (2004).

5. Stetz, J. B., Kendall, K. C. & Macleod, A. C. Black bear density in Glacier National Park, Montana. *Wildl. Soc. Bull.* **38**, 60–70 (2014).

6. Kunkel, K. E., Ruth, T. K., Pletscher, D. H. & Hornocker, M. G. Winter prey selection by wolves and cougars in and near Glacier National Park, Montana. *J. Wildl. Manage.* **63**, 901–910 (1999).

7. Kendall, K. C. *et al.* Grizzly bear density in Glacier National Park, Montana. *J. Wildl. Manage.* **72**, 1693–1705 (2008).

8. Tomson, S. D. Ecology and summer/fall habitat selection of American marten in northern Idaho. (University of Montana, 1999).

9. Mitchell, J. L. Mink movements and populations on a Montana river. *J. Wildl. Manage.* **25**, 48–54 (1961).

10. Perrine, J. D. Ecology of Red Fox ( Vulpes vulpes ) in the Lassen Peak Region of California , USA. (University of California, Berkeley, 2005).

11. Melquist, W. E. & Hornocker, M. G. Ecology of river otters in west central Idaho. *Wildl. Monogr.* **83**, 3–60 (1983).

12. Lofroth, E. C. & Krebs, J. The Abundance and distribution of wolverines in British Columbia, Canada. *J. Wildl. Manage.* **71**, 2159–2169 (2007).

13. Bekoff, M. & Gese, E. M. Coyote. in *Wild Mammals of North America: Biology, Management, and Conservation* (eds. Feldhamer, G., Thompson, B. & Chapman, J.) 467–481 (Johns Hopkins University Press, 2010).

14. Cheng, E., Hodges, K. E. & Mills, L. S. Impacts of fire on snowshoe hares in Glacier National Park, Montana, USA. *Fire Ecol.* **11**, 119–134 (2015).

15. Singer, F. J. & Mack, J. A. Potential ungulate prey for Gray Wolves. in *Ecological Issues on Reintroducing Wolves to Yellowstone National Park* (ed. Cook, R.) 75–117 (United States Department of Interior, National Park Service, 1993).

16. Smith, D. W., Peterson, R. O. & Houston, D. B. Yellowstone after wolves. *Bioscience* **53**, 330 (2003).

17. Singer, F. J. The ungulate prey base for wolves in Yellowstone National Park. in *The Greater Yellowstone Ecosystem: Redifining America’s Wilderness Heritage* (eds. Keiter, R. B. & Boyce, M. S.) 323–348 (Yale University Press, 1991).

18. Messick, J. P. & Hornocker, M. G. Ecology of the badger in southwestern Idaho. *Wildl. Monogr.* 3–53 (1981) doi:10.1002/jwmg.ll3.

19. Smith, D. S. Habitat use home range and movements of bobcats in western Montana. (University of Montana, 1984).

20. Crabtree, R. L. & Sheldon, J. W. The ecological role of coyotes on Yellowstone’s northern range. *Yellowstone Sci.* 15–23 (1999).

21. Vonholdt, B. M. *et al.* The genealogy and genetic viability of reintroduced Yellowstone grey wolves. *Mol. Ecol.* **17**, 252–274 (2008).

22. Schwartz, C. C. *et al.* Temporal, spatial, and environmental influences on the demographics of grizzly bears in the Greater Yellowstone Ecosystem. *Wildl. Monogr.* **161**, 1–68 (2006).

23. Clark, T. W., Campbell, T. M. I. & Hauptman, T. N. Demographic characteristics of American marten populations in Jackson Hole, Wyoming. *Gt. Basin Nat.* **49**, 587–596 (1989).

24. Quigley, H. & Hornocker, M. G. Cougar population dynamics. in *Cougar Ecology and Conservation* (eds. Hornocker, M. G. & Negri, S.) 59–75 (University of Chicago, 2010).

25. Pengeroth, D. Characteristics of a striped skunk population in the Mission Valley Montana. (University of Montana, 1991).

26. Hodges, K. E., Mills, L. S. & Murphy, K. M. Distribution and abundance of snowshoe hares in Yellowstone National Park. *J. Mammal.* **90**, 870–878 (2009).

27. Flinders, J. T. & Chapman, J. A. Black-tailed Jackrabbit. in *Wild Mammals of North America: Biology, Management, and Conservation* (eds. Feldhamer, G., Thompson, B. & Chapman, J.) 126–146 (Johns Hopkins University Press, 2010).

28. Jenkins, K. J. & Starkey, E. E. *Influences of adjacent forest management activities on migratory elk of Mount Rainier National Park*. (1990).

29. Gaines, W. L., Lyons, A. L., Lehmkuhl, J. F. & Raedeke, K. J. Landscape evaluation of female black bear habitat effectiveness and capability in the North Cascades, Washington. *Biol. Conserv.* **125**, 411–425 (2005).

30. Lewis, J. C. & Stinson, D. W. *Washington State Status Report for the Fisher*. (1998).

31. Romain-Bondi, K. A. *et al.* Density and population size estimates for North Cascade grizzly bears using DNA hair-sampling techniques. *Biol. Conserv.* **117**, 417–428 (2004).

32. Taper, M. L., Meagher, M. & Jerde, C. L. *The phenology of space: spatial aspects of bison density dependence in Yellowstone National Park*. http://pubs.er.usgs.gov/publication/70159669 (2000).

33. White, P. J., Davis, T. L., Barnowe-Meyer, K. K., Crabtree, R. L. & Garrott, R. A. Partial migration and philopatry of Yellowstone pronghorn. *Biol. Conserv.* **135**, 502–510 (2007).

34. Sawyer, H., Lindzey, F. & McWhirter, D. Mule deer and pronghorn migration in western Wyoming. *Wildl. Soc. Bull.* **33**, 1266–1273 (2005).

35. Gehrt, S. D. Raccoon. in *Wild Mammals of North America: Biology, Management, and Conservation* (eds. Feldhamer, G. A., Thompson, B. C. & Chapman, J. A.) 611–634 (Johns Hopkins University Press, 2010).

36. McKay, D. O. & Verts, B. J. Estimates of some attributes of a population of Nuttall’s cottontails. *J. Wildl. Manage.* **42**, 159–168 (1978).

37. Rosatte, R. & Lariviere, S. Mink. in *Wild Mammals of North America: Biology, Management, and Conservation* (eds. Feldhamer, G. A., Thompson, B. C. & Chapman, J. A.) 692–707 (Johns Hopkins University Press, 2010).

38. Koehler, G. M. Population and habitat characteristics of lynx and snowshoe hares in north central Washington. *Can. J. Zool.* **68**, 845–851 (1990).

39. Fuller, T. K. & Keith, L. B. Woodland caribou population dynamics in northeastern Alberta. *J. Mammal.* **45**, 197–213 (1981).

40. Belt, J. J. & Krausman, P. R. Evaluating population estimates of mountain goats based on citizen science. *Wildl. Soc. Bull.* **36**, 264–276 (2012).

41. Boyd, D. K., Ream, R. R., Pletscher, D. H. & Fairchild, M. W. Prey taken by colonizing wolves and hunters in the Glacier National Park area. *J. Wildl. Manage.* **58**, 289–295 (1994).

42. Myers, P. *et al.* The Animal Diversity Web. https://animaldiversity.org (2020).

43. Commission for Environmental Cooperation. *North America Environmental Atlas Land Cover 30m (Landsat and RapidEye)*. http://www.cec.org/north-american-environmental-atlas/land-cover-30m-2015-landsat-and-rapideye/ (2015).

44. Millar, J. S. & Zammuto, R. M. Life histories of mammals: an analysis of life tables. *Ecology* **64**, 631–635 (1983).

45. Oakleaf, J. K. *et al.* Habitat selection by recolonizing wolves in the northern Rocky Mountains of the United States. *J. Wildl. Manage.* **70**, 554–563 (2006).

46. Cushman, S. A., McKelvey, K. S. & Schwartz, M. K. Use of empirically derived source-destination models to map regional conservation corridors. *Conserv. Biol.* **23**, 368–376 (2009).

47. Schwartz, M. K. *et al.* Wolverine gene flow across a narrow climatic niche. *Ecology* **90**, 3222–3232 (2014).

48. McKelvey, K. S. *et al.* Climate change predicted to shift wolverine distributions, connectivity, and dispersal corridors. *Ecol. Appl.* **21**, 2882–2897 (2011).

49. Carroll, C., Mcrae, B. H. & Brookes, A. Use of linkage mapping and centrality analysis across habitat gradients to conserve connectivity of gray wolf populations in western North America. *Conserv. Biol.* **26**, 78–87 (2012).

50. Parks, S. A., McKelvey, K. S. & Schwartz, M. K. Effects of weighting schemes on the identification of wildlife corridors generated with least-cost methods. *Conserv. Biol.* **27**, 145–154 (2013).

51. Peck, C. P. *et al.* Potential paths for male-mediated gene flow to and from an isolated grizzly bear population. *Ecosphere* **8**, e01969 (2017).

52. Singleton, P. H., Gaines, W. L. & Lehmkuhl, J. F. *Landscape permeability for large carnivores in Washington: A geographic information system weighted-distance and least-cost corridor assessment*. (2002).

53. Long, R. A. *et al.* *The Cascades Carnivore Connectivity Project: A Landscape Genetic Assessment of Connectivity in Washington’s North Cascades Ecosystem. Final Report for the Seattle City Light Wildlife Research Program.* (2013).

54. Bailey, V. & Bailey, F. M. *Wild Animals of Glacier National Park.* (Washington D.C. Printing Office, 1918).

55. Bailey, V. *Animal Life of Yellowstone National Park*. (Thomas Publisher, 1930).

56. Banfield, A. W. F. The mammals of Waterton Lake National Park, Alberta, Canada. *Wildl. Manag. Bull. Ser. No. 1. Can. Wildl. Serv.* (1947).

57. Wright, P. L. Check list of the recent mammals of Montana. *Proc. Mont. Acad. Sci.* **10**, 47–50 (1951).

58. Negus, N. C. & Findley, J. S. Mammals of Jackson Hole, Wyoming. *J. Mammal.* **40**, 371–381 (1959).

59. Long, C. A. The mammals of Wyoming. *Uni. Kans. Publ. Mus. Nat. Hist* **14**, 493–758 (1965).

60. Hoffman, R. S., Wright, P. L. & Newby, F. E. The distribution of some mammals in Montana. I. Mammals other than bats. *J. Mammal.* **50**, 579–604 (1969).

61. Lechleitner, R. R. Mammals of Glacier National Park. *Glacier Nat. Hist. Assoc. Bull. No. 6.* (1973).

62. Taylor, W. P. & Shaw, W. T. *Mammals and Birds of Mount Rainier National Park*. (U.S. Govt. Printing Office, 1927).

63. Dalquest, W. W. Mammals of Washington. *Uni. Kans. Publ. Mus. Nat. Hist.* **2**, 1–444 (1948).

64. Carl, G. C., Guiguet, C. K. & Hardy, G. A. A natural history survey of the Manning Park Area, British Columbia. *Occas. Pap. Brit. Colum. Prov. Mus. No. 9* (1952).

65. Schamberger, M. L. *The mammals of Mount Rainier National Park*. (Oregon State University, 1970).

66. Weisbrod, A. R. Insularity and mammal species number in two national parks. in *Proceedings of First Conference on Scientific Research in the National Parks. Vol. 1.* (ed. Linn, R. M.) 83–88 (U.S. Government Printing Office, 1976).

67. Dragavon, J. A. Distribution of Small Mammals in Mount Rainier National Park. (University of Washington, Seattle, 1978).

68. Laufer, J. R. & Jenkins, P. T. *A preliminary study of gray wolf history and status in the region of the Cascade Mountains of Washington State*. (1989).

69. Johnson, R. E. & Cassidy, K. M. *Terrestrial mammals of Washington State: location data and predicted distributions. Vol 3.* (1997).
